# Validation of a measure of parental responsiveness: Comparison of the brief Parental Responsiveness Rating Scale with a detailed measure of responsive parental behaviours

**DOI:** 10.1177/1367493521996489

**Published:** 2021-02-25

**Authors:** Sarah Ellen Barnett, Penny Levickis, Cristina McKean, Carolyn Letts, Helen Stringer

**Affiliations:** 1 School of Education, Communication & Language Sciences, 5994 Newcastle University, Newcastle upon Tyne, UK; 2 34361 Murdoch Children’s Research Institute, Melbourne, Victoria, Australia; 3 Melbourne Graduate School of Education, The University of Melbourne, Melbourne, Victotia, Australia

**Keywords:** Parental behaviour, child, preschool, community health nursing, language development, observational methods, scales

## Abstract

Parental responsiveness is vital for child language development. Its accurate measurement in clinical settings could identify families who may benefit from preventative interventions; however, coding of responsiveness is time-consuming and expensive. This study investigates in a clinical context the validity of the Parental Responsiveness Rating Scale (PaRRiS): a time- and cost-effective global rating scale of parental responsiveness. Child health nurse (CHN) PaRRiS ratings are compared to a detailed coding of parental responsiveness. Thirty parent–child dyads completed an 8-min free-play session at their 27-month health review. CHNs rated the interaction live using PaRRiS. Videos of these interactions were then blindly coded using the more detailed coding system. PaRRiS ratings and detailed codings were compared using correlational analysis and the Bland–Altman method. PaRRiS and the detailed coding showed a moderate-strong correlation (*rs* (28) = 0.57, 95% CI [0.26, 0.77]) and high agreement (Bland–Altman). CHNs using PaRRiS can capture parental responsiveness as effectively as trained clinicians using detailed coding. This may allow (1) increased accuracy and efficiency in identifying toddlers at risk for long-term language difficulties; (2) more accurate allocation to speech and language therapy (SLT) services; (3) decreased burden on SLT resources by empowering CHNs to make more informed referral decisions.

## Introduction

Between 7% and 14% of children have language difficulties in the early years (0–5), and approximately 7.6% have difficulties which persist into the school years (Law et al., 2017; Norbury et al., 2016; Tomblin et al., 2003). With substantially increased risk of poor educational, behavioural and psychosocial outcomes for untreated difficulties (Bercow, 2008; Snowling et al., 2001, 2006), early identification of children at risk of long-term language difficulties is crucial; however, identification of these children is problematic.

Recent population-based studies have demonstrated considerable variation in early language development: some children have a slow start which then resolves, some have persisting difficulties and some have difficulties which emerge later in their development (Law et al., 2017). This volatility means available language screening tools are not sufficient to identify children in need of intervention. Randomised controlled trials (RCTs) of parent–child interventions which have targeted families based only on toddler’s language abilities have found null effects. This is not necessarily because the children do not progress, but because control groups catch up equivalently to the intervention group (Wake et al., 2011). It has been suggested that combining a language screen with additional information on known risks could help identify children at risk for persistent problems. However, ‘traditional’ risk factors, such as socioeconomic status (SES) and family history of speech and language problems, only moderately predict language at 4 years (Law et al., 2012).

Parental responsiveness refers to the characteristics of a parent’s response to a child which is contingent on the child’s preceding behaviour (Eshel et al., 2006). A responsive parent is not directive of their child’s attention but follows their child’s lead, watching and listening carefully for communication. Responsiveness is directly related to child language levels from infancy and throughout the early years (Donnellan et al., 2019; Goldstein and Schwade, 2008; Hirsh-Pasek et al., 2015), and ‘there is no evidence that the value of adult responsiveness to child communicative initiations declines with age’ (Rowe and Snow, 2019: 8). Indeed, parental responsiveness is the subject of many effective parent–child interaction interventions (see Roberts and Kaiser, 2011 for a review). Because of its evident links with child language development, parental responsiveness has recently been investigated as a potential predictive risk factor in Western, English-speaking samples. Much of the parent–child interaction literature includes Western, English-speaking dyads, and it is therefore important to acknowledge that the same types of parenting behaviours and styles may not be acceptable or appropriate for families from other countries or with different cultural backgrounds. Levickis et al. (2014; 2018b) used a detailed coding scheme of four specific responsive parent behaviours (see Supplemental Material), in a parent–child play interaction: parent’s use of expansions (repeating and expanding on a child’s utterance), imitations (imitating a child’s utterance), responsive questions (wh- questions based on the child’s focus of attention) and labels (naming the child’s focus of attention). They found that higher frequency use of expansions, imitations and responsive questions predicted better child language outcomes at ages two, three and 4 years, while higher labelling predicted poorer language outcomes. The authors posit that increased parental labelling could be a reflection of a child’s language level; children with fewer words will afford less opportunity for parental expansions and imitations since these are dependent on a child using words, and labels will therefore occur more. This seems highly likely, given that there remains a wide body of evidence verifying that responsive labelling is positively linked to children’s language, particularly between 12 and 24 months (McGillion et al., 2017; Namy and Nolan, 2004; Tamis-LeMonda et al., 2001). Overall, there does appear to be a solid foundation for using parental responsiveness as a potential predictive factor for children’s language levels. Perhaps, if it were possible to identify parents clinically with more limited responsiveness, then interventions could be targeted at those families who would benefit most.

As part of the universal Healthy Child Programme in England, parents are offered regular health and development reviews from birth to around 27 months, which are typically carried out by child health nurses (CHNs) (also known as health visitors in the United Kingdom). The aim of the 27-month review is to optimise child development and well-being, and one of the domains included at this visit is speech and language development. Typically, by this age, a child can understand complex instructions, uses two-to-three word combinations, can be understood by those close to them and can produce a range of 200 or more words. If this is not the case, then a CHN may refer the child to audiology or speech and language therapy (SLT) if deemed appropriate. Qualitative findings from a recent study (Levickis et al., 2019) suggest that sometimes referrals from CHNs to SLT can occur too soon, so that by the time a child is seen, they have improved and no longer need SLT input. A CHN within the same study commented that by capturing a snapshot of parent–child interaction, they can determine what parents are/are not doing and can provide strategies to the parent so that they can support their child’s language development. A measure of parent–child interaction in addition to measures of child language ability could therefore help to gain a better understanding of what kind of communicative interactions are happening in the home environment and could be used to provide additional information which informs referral decisions to SLT.

Down et al. (2014) developed an easy-to-use rating scale of measuring parental responsiveness, the Parental Responsiveness Rating Scale (PaRRiS) (see Table 1), adapted from Marfo’s maternal responsiveness scale (Marfo, 1992: 224). PaRRiS was formed in response to the substantial limitations of the detailed coding scheme used in the aforementioned research by Levickis et al. (2014, 2018b). The scheme required around 60 minutes for a trained clinician to code 5 minutes of a parent–child interaction video using video coding software, meaning it is not viable for use in clinical settings. PaRRiS provides a global rating of responsiveness from 1 to 5, based primarily on how developmentally appropriate parents’ contributions are, and how directive they are of their child’s attention, the less directive (i.e. the more responsive), the better. Down and colleagues found PaRRiS was moderately correlated with the detailed coding of responsiveness (*r* (242) = 0.44, 95% CI [0.35, 0.60]. This means that PaRRiS could potentially be used clinically to measure parental responsiveness. If PaRRiS is to be used like this, however, further work is needed to test its validity in conditions which more closely resemble those in which it would be clinically applied than in studies conducted to date. Down et al. sampled mostly middle-upper SES families, involved only slow-to talk children, and interactions took place in the home with speech and language therapists (SLTs) rating the interaction via video. CHNs work most closely with toddlers and their parents in the United Kingdom and are vital in the identification process of children needing extra support from SLTs. Therefore in this study, we aim to explore the validation of PaRRiS when used by CHNs in a clinic setting, face to face with parents and their children, who are of mixed language ability and middle-lower SES.

**Table 1.** PaRRiS: the Parental Responsiveness Rating Scale (adapted from Marfo 1992: 224).

Rating	Definition
1 = very low	Mother rarely responds in a developmentally appropriate way either verbally or non-verbally to any of child’s gestures or verbalisations, and mother attempts to redirect child’s behaviour^a^, rather than following child’s interests.
2 = low	Mother responds occasionally in a developmentally appropriate way either verbally or non-verbally to child’s gestures or verbalisations, and/or mother spends more time attempting to redirect child’s behaviour than following child’s interest.
3 = moderate	Mother spends some time responding in a developmentally appropriate way either verbally or non-verbally to child’s gestures or verbalisations and sometime ignoring them, and/or mother spends equal time following child’s interest and redirecting child’s behaviour.
4 = high	Mother often responds in a developmentally appropriate way either verbally or non-verbally to child’s gestures or verbalisations, and/or mother spends more time following child’s interest than redirecting child’s behaviour.
5 = very high	Mother frequently responds in a developmentally appropriate way either verbally or non-verbally to child’s gestures or verbalisations, and mother does not attempt to redirect child’s focus from the current activity but follows child’s interests.

^a^‘Redirecting the child's behaviour’ refers to redirecting the child’s attention away from their current play and interests at that point in time.

## Aim

To validate PaRRiS as a global rating of parental responsiveness, by comparing it to the detailed coding of parent responsive behaviours (components of responsiveness) in a different context to that of the Down et al.’s study, and one that is ecologically valid. Addressing this aim will help to determine whether PaRRiS could be used in CHN’s practice to measure parental responsiveness. Given the remit of CHNs to provide universal preventative public health services to families of toddlers to improve their health and well-being, PaRRiS could be an invaluable addition to the CHN assessment and intervention ‘toolkit’ (Katz et al., 2010; Pring et al., 2012).

## Methods

### Study design

This cross-sectional observational study is nested within the IMPACT project (Levickis et al., 2018a), which aims to determine whether PaRRiS can be effectively and reliably used by healthcare and educational professionals to identify families most likely to benefit from parent-focused language interventions, and to explore families’ experiences of interventions. Ethical approval for the IMPACT project was obtained from the UK’s National Health Service (NHS) Health Research Authority (HRA #). Ethical approval was sought and obtained for the current sub-project from Newcastle University's Education, Communication and Language Sciences Ethics Committee.

### Recruitment

From September 2017 to June 2018, five CHNs were recruited to the IMPACT study led by the second author (A2), through an NHS Trust’s Community Matron and Health Visitor Area Leads in the North East of England. All CHNs provided written informed consent to participate. Between October 2017 and June 2018, these CHNs recruited 30 parents whose children were due for their 27-month health check. All families attending a centre for their child’s 27-month review (i.e. not those having reviews in their home) during this time were eligible to take part. The second author was available to go through the consent form verbally to ensure parents understood what was being asked of them. Parents were excluded if they were not able to understand the information sheet and consent form (*n* = 0), otherwise all parent–child dyads fitting the above criteria were eligible. All parents provided written informed consent for themselves and their child to participate, including consent for videos to be recorded and stored securely (*n* = 30).

### Procedures

#### Training

Once recruited, CHNs attended a workshop with A2, who is an expert in the detailed coding and was involved in the development of PaRRiS. CHNs were trained in the use of PaRRiS, using several practice training videos, until reaching 80% or greater reliability with A2’s ratings.

For the detailed coding of videos, the first author (A1) studied a manual for the detailed coding scheme created by Levickis et al. (2014) (see Supplemental Material), and coded three training videos using this on BORIS software (Friard and Gamba, 2016). This allows the user to organise, code and analyse observational data. Blinded inter-rater reliability between A1 and A2 was calculated on three training videos using Cohen’s kappa. Following precedent, a Cohen’s kappa score of 0.61 or greater was considered sufficient for agreement (Landis and Koch, 1977). This demonstrated appropriate knowledge of the detailed coding scheme for further analyses. Discussion between A1 and A2 clarified any disagreements after coding had taken place.

#### Data collection

A2 attended the 27-month health checks and set up a standard toy set and video recorder on a tripod. A2 then left the room to maintain blinding of ratings, and the CHNs observed in real time, the 8-minute parent–child free play of consenting dyads using PaRRiS rating form, assigning each of the 30 parents scores from 1 to 5. Parents then filled out a survey, including questions about their experience of the free-play session, general demographics, whether they had any concerns about their child’s language and whether their child was combining words. A2 rated the video recorded interactions post-visit using PaRRiS, blind to the CHN’s ratings. Any disagreement between A2’s and CHN’s PaRRiS ratings (*n* = 8, each by one point on the rating scale) was decided independently by A1, and these were the ratings used in the final analyses.

A1 coded 5 minutes of each of the 30 parent–child free-play videos in depth, using the detailed coding scheme. Because labelling remains an evidence-backed responsive behaviour, and because its precise mechanism with reference to child language is extraneous when comparing the two measures, it was retained in the detailed coding. Where possible, the 5-minute periods that were coded were taken from 1 minute into the video (*n* = 25) to allow for a warm-up period; hence an 8-minute play session was aimed for in filming. Five videos were coded from different start points for the following reasons: one video was coded from 2 minutes in as the child was out of camera shot before this, and four videos were coded from the beginning due to the length of the original videos being shorter than planned (i.e. less than 6 minutes). Video samples featuring both parents were rated by focusing on the parent most involved in the interaction. The presence of siblings in videos was considered to be representative of parent-child interactions at home and videos containing this were therefore included.

Detailed coding of the four responsive behaviours was completed using BORIS software (Friard and Gamba, 2016). The four responsive behaviours (expansions, imitations, responsive questions and labels) were all counted as types of verbalisation, and each were mutually exclusive. Any verbalisations which were not responsive behaviours were coded as ‘utterances’. Hence, data were coded as five types of verbalisation. Each video was watched four times and coded by A1. Responsive behaviours in each video were counted, and a ‘rate of frequency per minute’ was calculated for each.

#### Data analysis

Data analysis was completed in SPSS. To establish inter-rater reliability for the detailed coding, A2 completed detailed coding for 20% of the videos (*n* = 6), randomly selected and blind to A1’s coding. A Cohen’s kappa score of 0.61 or greater was considered sufficient for agreement, the same as for the training videos.

All data were tested for normality using the Shapiro–Wilk test. Depending on the normality of the distribution, a Pearson’s correlation coefficient or a Spearman’s rank-order correlation was used to determine the relationship between parental responsive behaviours (frequency of all responsive behaviour use per minute) and the CHN PaRRiS global rating of the parent-child interaction. The Bland–Altman method (also known as the Tukey mean-difference plot) is used widely to evaluate agreement between two measurement techniques or instruments. It allows for the identification of any outliers or any systematic difference between the measurements. It is a graphical method for comparing two measurement techniques, whereby the differences between the two measures are plotted against the averages of the two methods, and limits of agreement are constructed. These limits are calculated by using the mean and standard deviation of the differences between the two measures. Overall, the Bland–Altman method provides a measure of agreement that can be presented in conjunction with a correlation measurement. This is important, since high correlation does not necessarily imply good agreement between two measures (Bland and Altman, 1986). This method was used to indicate agreement between PaRRiS ratings and the detailed coding of responsiveness. The median and interquartile range of each individual responsive behaviour was also calculated.

## Results

### Reliability

Blinded inter-rater reliability between second and first authors' detailed coding was calculated on three training videos using Cohen’s kappa (κ = 0.71–0.87, (95% CI [0.48, 1.00]), percentage agreement 79–88%).

Blinded inter-rater reliability of A1’s coding of the video interactions using the detailed coding scheme showed moderate-strong agreement between the two raters’ judgements across all five types of utterance (κ = 0.91–1.00, (95% CI [0.85, 1.00]), percentage agreement 92–100%).

### Participants

Table 2 summarises the participant characteristics. The sample contained approximately equal numbers of boys and girls, and 24 (80%) parents completed additional education after the end of compulsory education at age 16 years. The Index of Multiple Deprivation (IMD) was used to measure SES. The IMD is a multifaceted measure of relative deprivation for small geographical areas in England. It takes into account multiple sets of data to provide an overall rank for deprivation. Factors include income, level of education and levels of crime in the area. In terms of the IMD, participants were on average slightly more disadvantaged than the general UK population, with a mean score of 3.03 (SD = 2.30) out of 10.

**Table 2.** Parent–child dyad characteristics and caregiver responsiveness ratings.

Characteristic	Total sample (*n* = 30)
Children	
Age at video recording (months), mean (SD)	27.8 (1.9)
Male sex % (*n*)	56.7 (17)
Hears non-English language regularly at home % (*n*)^a^	0.03 (1)
Diagnosis of developmental condition % (*n*)^a^	0.03 (1) (hearing loss)
Parents (1 per child)	
Education % (*n*)^b^	
Left full-time education at ≤16 years	20 (6)
Left full-time education at ≥17 years	80 (24)
IMD Index of Multiple Deprivation^c^ score/10% (*n*)	
1	50 (15)
4	27 (8)
5	13 (4)
8	10 (3)
Presence at free-play session % (*n*)	
Mother only	63 (19)
Father only	7 (2)
Mother and father	17 (5)
Other^d^	13 (4)
Focus of detailed coding % (*n*)	
Mother	80 (24)
Father	13 (4)
Other^e^	7 (2)
Parental responsiveness	
PaRRiS rating % (*n*)	
1 = very low	1 (3.3)
2 = low	5 (16.7)
3 = moderate	16 (53.3)
4 = high	8 (26.7)
5 = very high	0 (0)
Detailed rating median (interquartile range)	
Talkativeness (rate of utterance/minute)	13.5 (7.1)
Rating score (rate of all responsive behaviours/minute)	4.2 (3.5)
Overall utterances	67.5 (35.3)
Overall responsive behaviours	21 (17.5)

^a^Data available from 28 children.

^b^Data available from 27 parents.

^c^Measure of socioeconomic status. Derived from national census; English Index of Multiple Deprivation (rating 1–10; 1 being most deprived and 10 being least). No participants were in deciles 2, 3, 6, 7, 9 or 10.

^d^In some sessions, grandparent, foster parent and/or siblings were present.

^e^One grandmother and one foster mother.

On average, parents were rated ‘moderately’ responsive on PaRRiS (mean score 3.03 out of 5, SD = 0.77). The median score of responsive behaviours per minute was 4.2 with an interquartile range of 3.50. Responsive questions were the most used behaviour (mean 1.71 per minute), with labels being the least used (mean 0.77 per minute). Imitations were slightly more commonly used than expansions (mean 0.93 and 0.83 per minute, respectively). Further information on the distribution of individual responsive behaviours can be found in the Supplemental Material.

**Figure 1.**
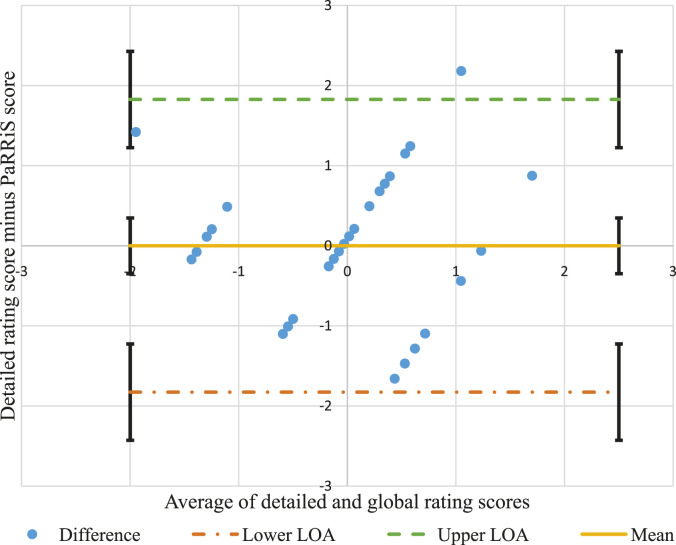
Bland–Altman plot showing the agreement between the detailed rating of caregiver responsiveness and the Parental Responsiveness Rating Scale (PaRRiS), with 95% limits of agreement (converted z-scores) and 95% confidence intervals.

A Spearman’s rank-order correlation (*r*_*s*_) showed a moderate-strong, positive correlation between the researcher’s detailed coding of responsiveness and CHN’s PaRRiS ratings (*r*_*s*_ = 0.57, 95% CI [0.26, 0.77]. This agreement was confirmed by the Bland–Altman method, see Figure 1, where all differences between the two ratings are within the upper and lower confidence levels of the limits of agreement, and thus, it can be said that the two ratings show acceptable agreement (Giavarina, 2015).

## Discussion

The findings of this study strengthen conclusions drawn from the results of Down et al.’s study (2014). They show that PaRRiS, a global measure of responsiveness used by CHNs, when compared with a detailed coding of responsive parent behaviours indicates a moderate-strong correlation and very high agreement. The correlation in conjunction with high agreement is a vital indication that these two measures are matched well. This is significant for two main reasons. Firstly, because PaRRiS explicitly considers parent directiveness where the detailed coding does this only implicitly, and secondly, because the measures differ so much in terms of time efficiency and expertise needed. The agreement between the two methods of measuring parental responsiveness shown here means that PaRRiS has the potential to be used by health professionals to capture parental responsiveness with children at 27 months in a quick and cost-effective manner.

This study has several implications for the development of clinical services for children who are slow to talk or at risk of poor language development. Firstly, it has been difficult to know whether early intervention is targeting the right groups, due to natural volatility of language in young children. This has meant that sometimes parents who are already very responsive to their child receive interventions which target increasing responsiveness, which they do not need. This is clearly a poor use of resources and could increase anxiety in families where the outcome is likely to be positive. In other cases, a ‘watch and wait’ approach has been applied when in fact the family may have needed or benefited from early intervention. By accurately and efficiently identifying dyads at risk through incorporating their PaRRiS rating into decision-making, families and children will be more likely to receive the care they individually require. Adding PaRRiS as a potential new tool in CHN’s ‘toolkit’ to do this, and not an obligatory part of assessment, would add the greatest benefit. It could be reserved for children about whom there is specific concern regarding language and communication and could work alongside other measures of child language skills already in use. This, critically, could provide valuable insight into care decisions without encroaching on the limited time CHNs have available. Secondly, low SES groups are often targeted for early intervention, despite the fact that the way in which SES operates in relation to child language outcomes is unclear. It is suggested in recent literature that the relationship between SES and child language outcomes is spurious, masking the association with more directly influential factors such as language environment and parental input (Letts, 2018; Rowe, 2008). Targeting according to responsiveness instead of SES is less stigmatising and potentially more likely to be accurate in identifying children most at risk. Finally, the fact that CHNs can be trained in first line of universal surveillance could also reduce the burden of inappropriate referrals currently placed on SLTs, and this initial ‘sifting’ process could produce more appropriate triaging. Though these implications need to be tested in research studies, the potential of PaRRiS is clear.

### Strengths and limitations

The participants, data collection context, and the practitioners in this study represent the normal practice of the services within which the data were collected, providing high ecological validity. High inter-rater reliability is shown between the authors’ coding in both training in detailed coding and coding agreement in the video recordings. The former allows us to have confidence in the findings of the study, and the latter demonstrates the clinical validity of PaRRiS. This study is also representative of a different demographic to previous studies in this area, being a sample of predominately low SES participants and including fathers, both less well-researched populations in this field. Nevertheless, data may be influenced by combining mothers and fathers in this study, since their differences in play and interaction styles have commonly been reported (John et al., 2013; Lindsey and Caldera, 2006; Pancsofar and Vernon-Feagans, 2006). Although this may not have impacted on the key findings of the current study given we were comparing the ratings between use of PaRRiS and detailed rating, this should be taken into account when looking at overall responsiveness ratings and behaviours. Furthermore, the study sample size is small, (*n* = 30). These impacts on the generalisability of the findings and means confident claims cannot be made regarding its relationship with parental responsive behaviours (Levickis et al., 2014, 2018b). The sample is slightly skewed in terms of SES level, meaning it is potentially not representative of wider UK populations. Finally, more specific information about education levels and their relationship to the IMD data would have been helpful in understanding the sample more fully.

### Implications for practice

This study has shown that CHNs can objectively identify levels of parental responsiveness by using PaRRiS. This has the potential to improve efficiency of services by targeting appropriate support and intervention more accurately. Furthermore, timely referrals can be made to SLT services as the families in greater need can be identified at an earlier stage.

If surveillance programmes such as PaRRiS are to be worthwhile and indeed ethical, effective interventions must be developed. Evidence regarding parent–child interaction interventions is mixed. A crucial next step would be to determine whether PaRRiS identifies families who would benefit from interventions and to develop interventions tailored to the diverse needs of the populations served by CHNs and SLTs.

## Conclusion

PaRRiS shows very good agreement with a detailed coding method within a real-life setting. In combination with previous and concurrent investigations (Down et al., 2014; Levickis et al., 2019), this study shows that PaRRiS holds promise as a clinically applicable tool for use by CHNs to support them in their role of improving health and well-being outcomes in young children.

## Supplemental Material

sj-pdf-1-chc-10.1177_1367493521996489 – Supplemental Material for Validation of a measure of parental responsiveness: Comparison of the brief Parental Responsiveness Rating Scale with a detailed measure of responsive parental behaviours Click here for additional data file. Supplemental Material, sj-pdf-1-chc-10.1177_1367493521996489 for Validation of a measure of parental responsiveness: Comparison of the brief Parental Responsiveness Rating Scale with a detailed measure of responsive parental behaviours by Sarah Ellen Barnett, Penny Levickis, Cristina McKean, Carolyn Letts and Helen Stringer in Journal of Child Health Care

sj-pdf-2-chc-10.1177_1367493521996489 – Supplemental Material for Validation of a measure of parental responsiveness: Comparison of the brief Parental Responsiveness Rating Scale with a detailed measure of responsive parental behaviours Click here for additional data file. Supplemental Material, sj-pdf-2-chc-10.1177_1367493521996489 for Validation of a measure of parental responsiveness: Comparison of the brief Parental Responsiveness Rating Scale with a detailed measure of responsive parental behaviours by Sarah Ellen Barnett, Penny Levickis, Cristina McKean, Carolyn Letts and Helen Stringer in Journal of Child Health Care
