# Supplementary material for: Validation of a measure of parental responsiveness: Comparison of the brief Parental Responsiveness Rating Scale with a detailed measure of responsive parental behaviours
Source: J Child Health Care. 2021 Feb 25;26(1):56–67. doi: 10.1177/1367493521996489 (PMC8943477; doi:10.1177/1367493521996489)
Supplement: sj-pdf-1-chc-10.1177_1367493521996489 – Supplemental Material for Validation of a measure of parental responsiveness: Comparison of the brief Parental Responsiveness Rating Scale with a detailed measure of responsive parental behaviours [file sj-pdf-1-chc-10.1177_1367493521996489.pdf]

Detailed Coding of Parental Responsiveness

A manual to aid in the analysis of responsive behaviours in parent-child interactions

What is considered a verbalisation?

A verbalisation is any linguistic information directed at the child. It consists of a grammatical unit + breath unit + terminal intonation contour. In this coding scheme, all verbalisations were coded as either an Utterance (that is, a verbalisation which is not classed as a responsive behaviour), or a responsive behaviour: an Expansion, an Imitation, a Responsive Question or a Label.

The five types of verbalisation are mutually exclusive meaning that only one code may be assigned to a parental verbalisation. In some cases, parents used several verbalisations in a row. To address this situation, a hierarchy of codes was incorporated into the coding scheme so that it was clear which verbalisation should be coded. For example in a series of parental verbalisations whereby a parent says a string of sentences without any definite pauses, it was decided that only the first verbalisation would be coded and the rest would be ignored. Segmentation was based on a grammatical unit + breath unit + terminal intonation contour (i.e. the end of a pattern of pitch in speech). This hierarchy of codes was established as it was too difficult for a coder to retain all of the information from a series of verbalisations which could lead to confusion of which behaviours to code and this in turn could weaken reliability. An example of the hierarchy as it would be applied to a string of parental verbalisations is as follows:

> **E.g. 1 C:** *Drink.* **P:** *Drink.* [Imitation] (no breath pause or intonation contour) *Let's give baby a drink.* [Expansion] (no breath pause or intonation contour) *What is baby going to drink?* [Responsive Question].

This would be coded as an Imitation (as this is the first verbalisation).

> **E.g. 2 C:** *Dindin.* **P:** *Is it dindins time?* [Expansion] (no breath pause or intonation contour) *What are we having for dindins?* [Responsive Question].

This would be considered one verbalisation, and coded as an Expansion (as this is the first verbalisation).

> **E.g. 3 C:** *looking at 2 animals for Old MacDonald song* **P:** *Which one?* (no breath pause or intonation contour) *Are we going to do the cow?*

This would be considered one verbalisation, and would not be coded as a responsive behaviour, as the parent, by suggesting '*Are we going to do the cow?*' does not give chance for the child to respond, not making '*Which one?*' a Responsive Question.

> **E.g. 4 C:** *Where's Aunty Sarah?* **P:** *Can you see her?* (no breath pause or intonation contour) *What's she doing?*

In cases where the parent's immediate response would be classed as an utterance, as in this example, but is followed immediately by a responsive behaviour, the responsive behaviour **is counted**.

If there are clear breaks between as according to the segmentation guidelines, each is considered its own verbalisation e.g.:

> **C:** *Nana*. **P:** *Is it a banana?* [Expansion] (breath pause and intonation contour) *Who has the banana?* [Responsive Question].

This would be coded considered two verbalisations, and both Expansion and Responsive Question would be coded, as long as the child was still focusing on the banana when the Responsive Question was asked. However, if a parent corrects him/herself during a sentence so that there is a breath pause, this is considered one verbalisation, e.g.

> **P:** *do you want*- [breath pause] *do you want to put that in?*

Songs are coded as one verbalisation. However if they are broken up, they are coded each time the song restarts, and any Responsive Behaviours and Utterances between are coded as such, e.g.:

> **P:** *Old MacDonald had a farm eeiieeiioo, and on that farm he had* [Utterance]*pause* *What did he have?* [Responsive Question] *A sheep. With a baa baa here and a baa baa there…* [Utterance]

The only non-word vocalisations which are coded (and thus considered verbalisations) are:

- those which are Imitations of the child (can be anything including an intake of breath, or noises like 'urgh' or 'oh') which would *always* be coded as Imitations or Expansions (i.e. if these are not repetitions of the child they are not considered as verbalisations at all)
- OR vocalisations which have a specific linguistic meaning (e.g. animal/environment noises; 'uh huh' or 'mhmm' – meaning 'yes'; 'ah-ah' – meaning 'no'; 'Ah' or 'oh' – signalling comprehension; 'uh oh'; 'shh').

The following are examples of what would and would not be considered a verbalisation:

- ✓ **C:** *Intake of breath* **P:** *Intake of breath* [Imitation]
- ✓ **C:** *Makes toy person fall down stairs* **P:** Uh oh! [Utterance]
- ✗ **C:** Not me **P:** Oh *intake of breath* [neither considered a verbalisation]

Expansions

Expansions are coded when a parent repeats one or all of the child's preceding words and adds to the child's preceding verbalisation (Girolametto et al., 2002; Girolametto, Weitzman, Wiigs, & Pearce, 1999; Lasky & Klopp, 1982). This includes both simple and complex Expansions.

A **simple Expansion** is when a parent repeats immediately the child's preceding word approximation or verbalisation and completes the verbalisation by adding one or more morphemes (i.e. the smallest

meaningful unit of language, which may be a word or word element) or words. This includes any words, including praise, but excluding any form of ‘yes’.

- ✓ **C:** *Ball.* **P:** *It’s a red ball.*
- ✓ **C:** *One more.* **P:** *Another one.*
- ✓ **C:** *Frog.* **P:** *Frog, yeah, good girl.*

A **complex Expansion** is when a parent *repeats* the child’s preceding vocalisation/word *and adds* a question *within the same verbalisation*. This includes both yes/no questions and Responsive Questions.

- ✓ **C:** *Dirt.* **P:** *Dirt. What’s growing in the dirt?*
- ✓ **C:** *No.* **P:** *No, you don’t want a dummy do you?*
- ✓ **C**: *In there.* **P:** *In there. In where?*

If the parent does not repeat the verbalisation first, as in the examples above, and simply asks a question, this is coded differently. If this is a yes/no question, it is coded as a simple Expansion. If it is a Responsive Question, this is coded as a Responsive Question rather than an Expansion.

- ✓ **C:** *Pig.* **P:** *Is it a pig?* → **(Simple) Expansion**
- ✗ **C:** *Dog.* **P:** *What’s dog doing?* → **Responsive Question**

Imitations

An Imitation is coded when a parent repeats the child’s preceding vocalisation or verbalisation <u>exactly</u> or with a reduction of words (Girolametto et al., 2002; Girolametto et al., 1999; Lasky & Klopp, 1982). Imitations are also coded if there is an exact repetition of the child’s preceding verbalisation or vocalisations and an addition of any form of ‘yes’ (e.g. yes, yeah, mhmm). N.B. reductions such as from ‘piggy to ‘pig’ are still considered Imitations.

- ✓ **C:** *Bottle.* **P:** *Bottle.*
- ✓ **C:** *Mummy let’s go.* **P:** *Let’s go.*
- ✓ **C:** *Piggy.* **P:** *Pig, yeah.*
- ✓ **C:** *Oh.* **P:** *Oh.*

Responsive Questions

A Responsive Question is coded when the parent asks a ‘wh’ question (including ‘what’, ‘when’, ‘why’, ‘where’, ‘which’ ‘who’ ‘how’ and choice questions) that is *immediate* and *dependent on the child’s preceding act* (Tamis-LeMonda, Bornstein, & Baumwell, 2001), and which is intended to *require an answer*. The parent then waits (even briefly) for a response. The question can be about an object, event or activity, and can be phrased with ‘wh’ word at different points in the sentence. Requests for repetition, e.g. if the parent has not understood, and makes a request which is the equivalent of ‘What did you say?’ is coded as a responsive question as it is a direct question about the child’s preceding act (their verbalisation). Yes/no questions are not coded.

- ✓ **C:** *Daddy?* **P:** *What?*
- ✓ **C:** *Looking in bag* **P:** *Who's in the bag?*
- ✓ **C:** *Child holding a horse* **P:** *What's that?*
- ✓ **C:** *Child playing with toy train* **P:** *What does the train say?*
- ✓ **C:** *Oh dear.* **P:** *Oh dear what?*
- ✓ **C:** *In there.* **P:** *In where?*
- ✓ **C:** *Child attending to baby and bottles* **P:** *Look, how many bottles is there?*
- ✓ **C:** *unintelligible* **P:** *Sorry?*

Labels

A Label is coded when a parent Labels an object or action, *which is the focus of the child*, with the Label in the final position of the carrier phase (Girolametto et al., 2002; Girolametto et al., 1999; Tomasello & Farrar, 1986). Counting objects is not considered Labelling, however Labelling colours would be.

- ✓ **C:** *playing with toy horse* **P:** *That's a horse.*
- ✓ **C:** *Playing with toy stove* **P:** *Cooking.*
- ✓ **C:** *****Looks at mat* **P:** *What colour is this mat?* [Responsive Question] **C:** *no response, but continues attending to mat and parent* **P:** *It's green.* [Label]

Labels are not coded when they are phrased as a question:

- ✗ **C:** *Looking at pigs in book* **P:** *Are they pigs?*

If a parent Labels the focus of the child's attention after a child's verbalisation, and the child's verbalisation is unintelligible, mark as a Label. However if the verbalisation appears to be known (i.e. child says it consistently, or parent responds in such a way that the verbalisation appears to be something that the child says consistently), mark as Expansion or Imitation (depending on what the parent says).

References

Girolametto, L., Bonifacio, S., Visini, C., Weitzman, E., Zocconi, E., & Pearce, P. S. (2002). Mother-child interactions in Canada and Italy: Linguistic responsiveness to late-talking toddlers. International Journal of Language and Communication Disorders, 37(2), 153-171.

Girolametto, L., Weitzman, E., Wiigs, M., & Pearce, P. S. (1999). The Relationship between Maternal Language Measures and Language Development in Toddlers with Expressive Vocabulary Delays. American Journal of Speech-Language Pathology, 8(4), 364-374.

Lasky, E. Z., & Klopp, K. (1982). Parent-Child Interactions in Normal and Language-Disordered Children. Journal of Speech and Hearing Disorders, 47(1), 7-18.

Tamis-LeMonda, C. S., Bornstein, M. H., & Baumwell, L. (2001). Maternal responsiveness and children's achievement of language milestones. Child Development, 72(3), 748-767.

Tomasello, M., & Farrar, M. J. (1986). Joint attention and early language. Child Development, 57(6), 1454-1463.
