# Supplementary material for: Validation of a measure of parental responsiveness: Comparison of the brief Parental Responsiveness Rating Scale with a detailed measure of responsive parental behaviours
Source: J Child Health Care. 2021 Feb 25;26(1):56–67. doi: 10.1177/1367493521996489 (PMC8943477; doi:10.1177/1367493521996489)
Supplement: sj-pdf-2-chc-10.1177_1367493521996489 – Supplemental Material for Validation of a measure of parental responsiveness: Comparison of the brief Parental Responsiveness Rating Scale with a detailed measure of responsive parental behaviours [file sj-pdf-2-chc-10.1177_1367493521996489.pdf]

Distribution of individual responsive behaviours

**Responsive behaviour (rate per min)**		
Expansion, mean (SD) [median, IQR]	0.833 (0.86)	[0.5, 1.35]
Imitation, mean (SD) [median, IQR]	0.927 (0.83)	[0.8, 1.15]
Responsive Question, mean (SD) [median, IQR]	1.707 (1.26)	[1.4, 1.9]
Label, mean (SD) [median, IQR]	0.773 (0.80)	[0.6, 0.8]
